# Is senescence-associated β-galactosidase a marker of neuronal senescence?

**DOI:** 10.18632/oncotarget.12752

**Published:** 2016-10-19

**Authors:** Malgorzata Piechota, Piotr Sunderland, Adrianna Wysocka, Maria Nalberczak, Malgorzata A. Sliwinska, Kasia Radwanska, Ewa Sikora

**Affiliations:** ^1^ Laboratory of Molecular Bases of Aging, Nencki Institute of Experimental Biology, Polish Academy of Sciences, Warsaw 02-093, Poland; ^2^ Laboratory of Molecular Basis of Behavior, Nencki Institute of Experimental Biology, Polish Academy of Sciences, Warsaw 02-093, Poland; ^3^ Laboratory of Preclinical Studies in Neurodegenerative Diseases, Nencki Institute of Experimental Biology, Polish Academy of Sciences, 02-093, Warsaw, Poland

**Keywords:** aging, DNA damage response, neurons, senescence, SA-β-galactosidase

## Abstract

One of the features of cellular senescence is the activity of senescence-associated- β-galactosidase (SA-β-gal). The main purpose of this study was to evaluate this marker of senescence in aging neurons. We found that cortical neurons exhibited noticeable SA-β-gal activity quite early in culture. Many SA-β-gal-positive neurons were negative for another canonical marker of senescence, namely, double-strand DNA breaks (DSBs). Moreover, DDR signalling triggered by low doses of doxorubicin did not accelerate the appearance of neuronal SA-β-gal. *In vivo*, we observed pronounced induction of SA-β-gal activity in the hippocampus of 24-month-old mice, which is consistent with previous findings and supports the view that at this advanced age neurons developed a senescence-like phenotype. Surprisingly however, relatively high SA-β-gal activity, probably unrelated to the senescence process, was also observed in much younger, 3-month-old mice. In conclusion, we propose that SA-β-gal activity in neurons cannot be attributed uniquely to cell senescence either *in vitro or in vivo*. Additionally, we showed induction of REST protein in aging neurons in long-term culture and we propose that REST could be a marker of neuronal senescence *in vitro*.

## INTRODUCTION

Dividing cells inevitably face shortening of telomeres and activation of the DNA damage response (DDR) pathway upon each successive division until they become irreversibly growth-arrested. This phenomenon is known as replicative cell senescence. Senescence can also be induced prematurely by stress stimuli, which cause accumulation of double-strand DNA breaks (DSBs) - a process known as SIPS (stress-induced premature senescence) (reviewed in [1]). Accumulation of senescent cells has been discovered in aging organisms, e.g. in primates and humans [2, 3] and is considered as a cause of organismal aging [4]. Although non-proliferating, postmitotic cells, such as neurons, cannot be subject to replicative senescence, recently it has been suggested that activation of DDR and the appearance of certain cell senescence markers, such as the senescence associated-β-galactosidase (SA-β-gal) can be imputable to neuronal senescence [5].

Some research on neuron senescence has been carried out in long-term cultures of primary neurons and collected data seem to suggest that such cultures can be used as a model of neuronal aging [6, 7]. Overall, neurons can be kept alive for as long as 60 days, during which time, they display signs of development, maturation and finally aging and death. Initially, at 4 days *in vitro* (DIV) neurons have small bodies with very small number of neuritic outgrowths and later start to form a highly extensive network. Synaptogenesis lasts for the first 2-3 weeks. Deterioration of the neuritic network can be observed after 40DIV and aging is associated with such morphological changes as formation of varicosities along the processes and larger cell bodies. A question arises whether there are any changes in neurons during aging other than loss of dendrites and synapses. More specifically, can we find senescent markers in aging neurons and neurons undergoing DDR activation?

The most common marker of senescence is the lysosomal enzyme: Senescence-Associated β-galactosidase (SA-β-gal) measured at pH 6. Physiologically, β-gal is most active at low pH (pH 4), typical for lysosomes [8]. An increase in SA-β-gal was observed in brains of aging rodents [9]. It was shown that 24-month-old rats had more SA-β-gal in the hippocampus than young 6-month-old rats. Moreover, it was demonstrated that hippocampal and cerebellar neurons cultured *in vitro* also acquired SA-β-gal with time [9, 10]. Increased level of β- galactosidase was associated with brain aging in a work presented by Ori et al. [11]. However, in that case it is hard to determine in which cells- neurons, glia, blood wall cells or immune cells- this increase occurred. Interesting and comprehensive research done by von Zglinicki's group showed that neurons in aging brains developed a senescence-like phenotype, since they displayed SA-β-gal staining, γH2AX foci in their DNA, some heterochromatin foci, increased level of IL-6 and features of oxidative stress [5]. Furthermore, authors showed that in p21 knockout mice expression of these markers was reduced, based on which, they suggested that DDR activation was correlated with neuronal senescent state and that p21 was a necessary signal transducer between DDR and senescence in neurons.

This work has given positive answers to questions, whether senescent markers are present in aging neurons and neurons undergoing DDR activation.

In this study we present research performed on neuronal SA-β-gal and DDR in neuronal long-term cultures as well as in aging brains.

## RESULTS

### Induction of REST in long-term neuronal cultures

We used a common protocol for isolation of cortical neurons from rat embryos. Cortical neurons were maintained in culture for 30 days, which was based on numerous observations of their viability over time. Generally, viability varied between cultures and some of them displayed signs of deterioration (fragmented and bundled dendrites and condensing nuclei) before 30 DIV, whereas some looked viable and healthy at 30DIV.

At the beginning of culture we observed mainly neuronal cells identified by staining of MAP2 protein– a protein serving to stabilize microtubules growth uniquely in differentiated neurons. After about 10 DIV intense growth of glia, including astrocytes (GFAP-positive cells) and oligodendrocyte precursors (Olig2-positive cells), started (Supplementary Figure S1). Their number varied depending on the culture, however, at 30 DIV they usually did not exceed 30% (not shown). As we were interested in spontaneous and induced DNA lesions in neurons, we decided not to use a common protocol with cytosine arabinoside treatment for clearing the culture from non-neuronal cells, because cytosine arabinoside is known to induce DNA damage. Moreover, neuronal cells grow better in the microenvironment created by glial cells. Accordingly, to identify neurons or proteins of interest in neuronal cells, immunostaining of MAP2 was always performed.

**Figure 1:**
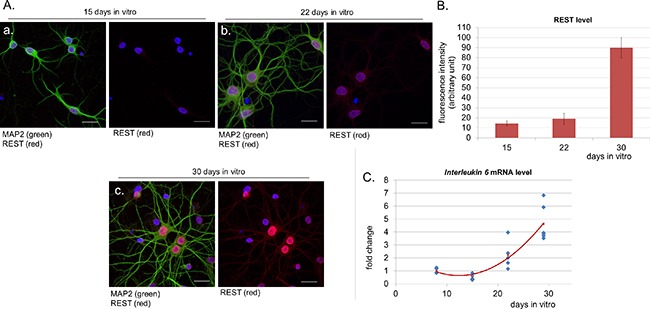
Nuclear level of REST increases in aged neurons in parallel to *IL-6* expression **A.** Confocal immunofluorescence labelling for REST (red), the neuronal marker MAP2 (green) and DNA (Hoechst, blue) in cortical neurons at 15 (a) 22 (b) and 30 DIV (days *in vitro*) (c). Scale bars represent 25 μm. **B.** Measurement of fluorescence intensity of nuclear REST in neurons. Data were obtained from three independent cultures. The majority of neurons stained positively for REST. **C.** Measurement of *IL-6* mRNA expression in neuronal cultures over time.

Aging is a multifactorial process associated with some specific transcriptional changes [11]. One of the most recently identified markers of neuronal aging is a transcription factor REST (ang. *repressor element 1-silencing transcription factor*) - a protein with neuroprotective properties, whose expression is low in the brain of young individuals and increases in older people [12]. It is known that aging neurons need REST to be protected from oxidative stress and toxic insults. In order to mark the aging stage in our neuronal cultures, we stained them with antibodies against this protein and observed a very intensive nuclear signal of REST in cultures at 30 DIV (Figure 1A, 1B).

### IL-6 induction in long-term neuronal cultures

We were interested if we could correlate upregulation of neuronal REST with other age-dependent changes occurring in our long-term cultures. It is known that brain aging is associated with increase in the level of IL-6 (interleukin 6) [13]. This pro-inflammatory cytokine is also secreted by senescent cells along with other factors which are collectively referred to as senescence-associated secretory phenotype (SASP) [14]. We measured *IL-6* mRNA level over time and observed that it increased reaching almost 5 times higher values at 29 DIV, when compared to 8 DIV (Figure 1C).

### SA-β-gal activity in neurons in long-term neuronal cultures

**Figure 2:**
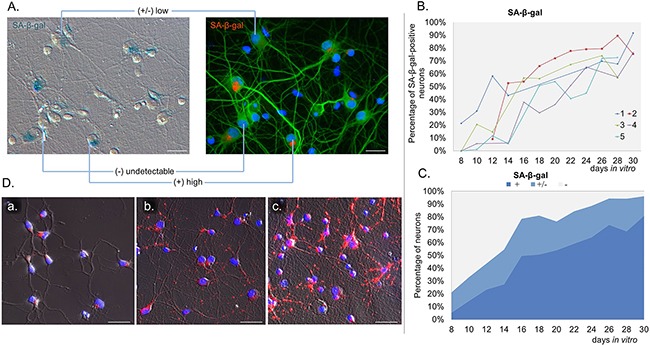
Gradual SA-β-gal induction in long-term neuronal cultures **A.** The image of neuronal staining (MAP2, green, right) merged with transmitted light image (left) of SA-β-gal activity (red) showing variability in the intensity of the SA-β-gal signal in neurons. **B.** Quantification of the percentage of neurons with high SA-β-gal signal in five cultures over a period of time. For each culture about 60 neurons were analysed per one time point. **C.** Mean values of the percentage of neurons exhibiting SA-β-gal activity with time: (+): high, (+/-): low, (-): undetectable SA-β-gal. **D.** Representative fluorescent images of LysoTracker Red staining in neuronal cultures over time: 4 DIV (a), 11 DIV (b), 18 DIV (c) from two independent experiments. Scale bars represent 25μm.

**Figure 3:**
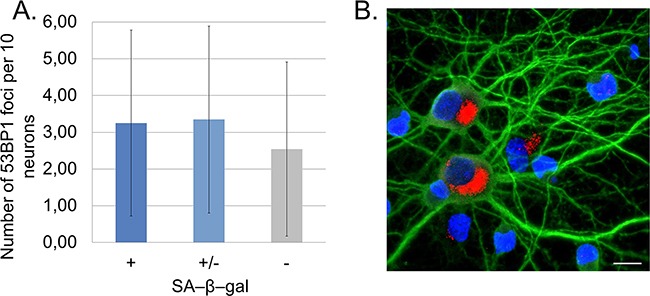
SA-β-gal-positive neurons do not accumulate DSB foci marked by p53BP1 staining **A.** Quantitative analysis of the number of p53BP1 foci in SA-β-gal-positive and negative neurons calculated from four cultures. At least 200 neurons were analysed in each group. Values represent the mean ± S.D. SA-β-gal (+): high, (+/-): low, (-): undetectable. **B.** A representative image of SA-β-gal-positive neurons (red) with no p53BP1 foci detected. A scale bar represents 10 μm.

**Figure 4:**
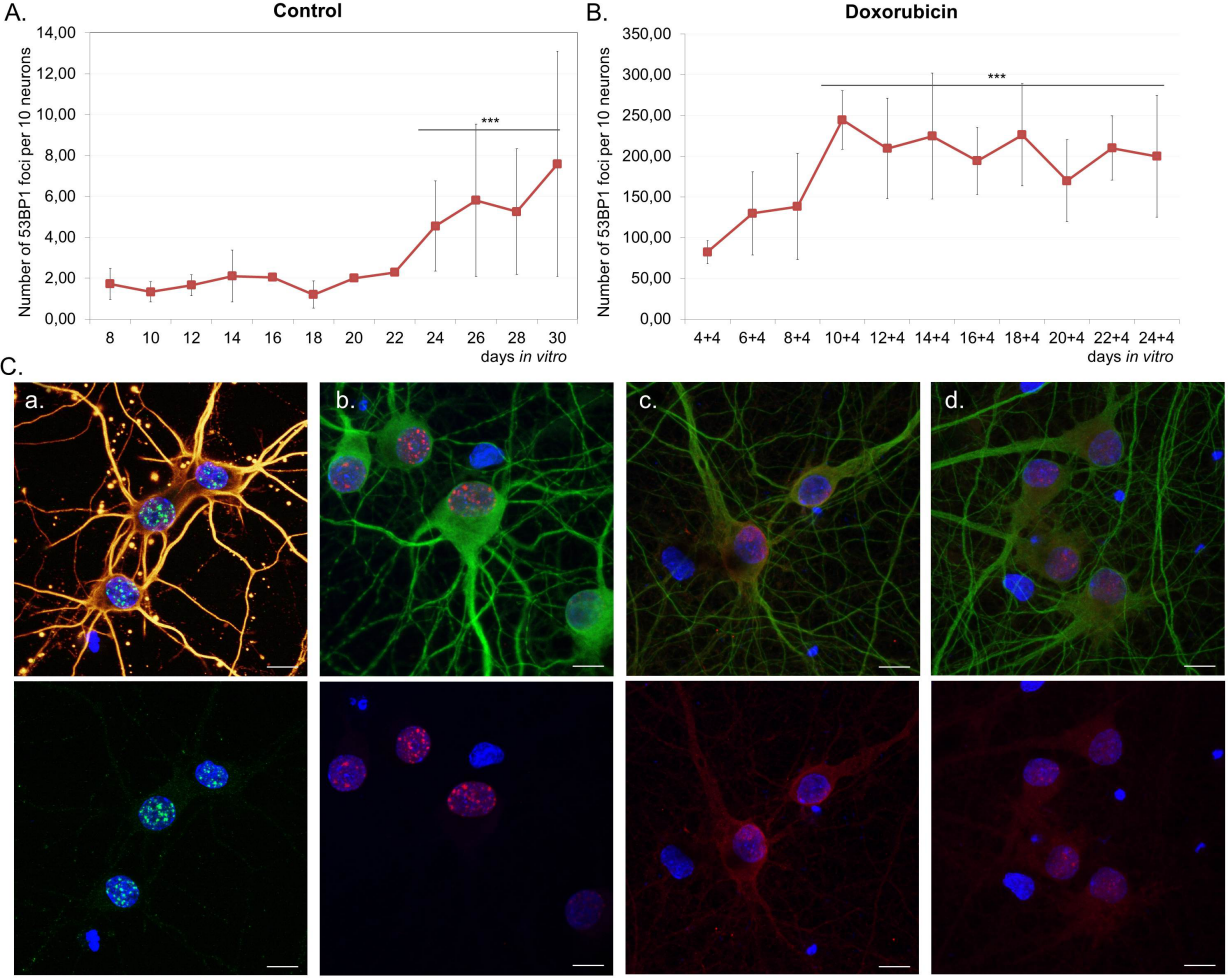
The number of DSB foci and DDR activation in long-term- and doxorubicin-treated neuronal cultures Neurons were cultivated untreated **A.** or treated with 10 nM doxorubicin for four days **B.** and stained for p53BP1. The average number of nuclear p53BP1 foci per ten neurons was calculated. For each culture at least 60 neurons were analysed at each time point. Values represent the mean ± S.D, ***P>0.001 by ANOVA from four independent experiments (control) and three independent experiments (doxorubicin); the mean value of 24-30 DIV relative to 8 DIV (for A), relative to 4+4 DIV (for B). **C.** DDR activation in 14 DIV neurons treated with doxorubicin. Images show staining for γH2AX (green), β-tubulin (yellow) (a), p53BP1(red), MAP2 (green) (b), P-ATM (Ser1981) (red), MAP2 (green) (c), P-CHK2 (Thr68) (red), MAP2 (green) (d). Scale bars represent 10 μm.

*In vitro* experiments allowed us to study changes at a single cell level. Because of the presence of glia in the cultures, we applied a novel method, which enabled us to detect SA-β-gal specifically in neurons. In this method a standard SA-β-gal staining is followed by immunofluorescence labelling of the neuronal marker MAP2. In this way, we were perfectly capable of identifying and quantifying of SA-β-gal-positive neurons (Figure 2A). Cells varied in terms of the intensity of the SA-β-gal signal. Some were completely deprived of the enzyme activity, while others were slightly or strongly positive. Based on the intensity of the SA-β-gal signal we divided cells into 3 groups: with undetectable-, low-, or high SA-β-gal. We studied five different cultures and all of them showed an increase of this common senescence marker over a period of time (high SA-β-gal), but at a slightly different rate for each culture (Figure 2B). It was observed that in all 30 DIV cultures about 80% of neurons had high SA-β-gal (Figure 2C). Surprisingly, we could observe SA-β-gal-positive neurons as early as at 8 DIV (in fairly young neurons). Interestingly, we also observed gradual increase in the lysosomal mass in live neuronal cultures stained with LysoTracker Red (Figure 2D). Therefore, we think that the increase in SA-β-gal is correlated with increase in the lysosomal number. A triple staining for SA-β-gal, MAP2 and p53BP1(a protein specifically binding DNA DSBs) revealed that SA-β-gal-positive neurons did not contain more DSBs than SA-β-gal-negative ones (Figure 3A). Furthermore, there were neurons which did not have any visible p53BP1 foci, in spite of being SA-β-gal-positive (Figure 3B). This means that the two main markers of cell senescence can arise independently. Interestingly, we observed a gradual, very slow increase in the number of DSBs foci with neuronal aging (Figure 4A). Generally, we observed a very low level of p53BP1 foci in neurons. Until 22 DIV the mean number of foci per ten neuronal cells was 2, but then increased approximately four times at 30DIV.

### SA-β-gal activity in neurons upon DDR activation

In subsequent experiments we tested the hypothesis that DDR activation might lead to increase in SA-β-gal activity in neurons. In order to induce DDR we treated cells with doxorubicin, which is a common DNA damaging agent and, depending on concentration, can be either cytotoxic or cytostatic. First, we determined neuronal survival upon doxorubicin treatment within a dose range between 10nM-1μM. The compound was highly cytotoxic to neuronal cultures at doses as low as 25nM, as revealed by the MTT assay (Supplementary Figure S2A). For further experiments we selected a 10nM dose, which resulted in about 40% loss of cell viability (Supplementary Figure S2B). This dose increased LDH release by about 50% (Supplementary Figure S2C) and caused apoptosis in 40% cells, as estimated by the number of condensing nuclei (Supplementary Figure S2D). We treated neuronal cultures with 10nM doxorubicin for four days and measured SA-β-gal activity and, additionally, the number of p53BP1 foci. Cultures were treated every two days until 24 DIV. In comparison with untreated cultures, we observed a substantial increase in the number of p53BP1foci in neurons (Figure 4B). Between 8-14 DIV the average number of foci per ten neurons increased from about 80 to more than 200 and then stabilized. Doxorubicin-treated cells at 14 DIV were checked not only for p53BP1, but also other DDR key proteins, namely γH2AX (phosphorylated H2AX histone), P-ATM (phosphorylated ataxia telangiectasia mutated protein) and P-CHK2 (phosphorylated checkpoint kinase 2). As can be seen in Figure 4C all of them were expressed implying DDR activation upon doxorubicin treatment.

**Figure 5:**
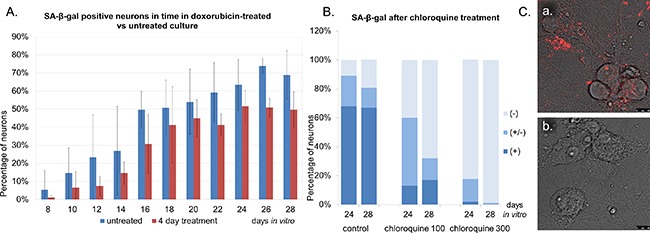
Doxorubicin-treated cultures do not have more SA-β-gal-positive neurons than untreated ones **A.** Neurons were treated with 10 nM doxorubicin for four days or cultivated untreated and stained for SA-β-gal. Quantitative analysis of the mean number of neurons with high SA-β-gal signal in untreated- versus doxorubicin-treated neurons. Values represent the mean ± S.D. from at least three independent experiments. **B.** A dose-dependent decrease in the number of SA-β-gal-positive neurons after chloroquine treatment. SA-β-gal (+): high, (+/-): low, (-): undetectable. **C.** A representative image of LysoTracker Red- stained neurons: control (a) or treated with chloroquine (b). Scale bars represent 10 μm.

Interestingly, our quantitative analysis indicated that the compound did not contribute to the increase in SA-β-gal activity (Figure 5A). Surprisingly, we observed even less high SA-β-gal-positive neurons in doxorubicin-treated versus untreated cultures. On average, doxorubicin-treated cultures had approximately 10-20% less SA-β-gal-positive neurons than untreated ones. However, because of various rates at which neurons from each culture acquired SA-β-gal, this difference was not statistically significant. What caused this apparent change in SA-β-gal? As it was already mentioned, β-galactosidase is a lysosomal enzyme and we suspected that this reduced SA-β-gal was a consequence of a specific activity of the drug on lysosomes. It has been published that doxorubicin can affect lysosomal morphology and function in mouse heart [15] and that it can localize to lysosomes in breast cancer cells [16]. We suggest that it may cause damage or dysfunction of neuronal lysosomes resulting in the drop of SA-β-gal activity. That SA-β-gal staining may be sensitive to the quality of lysosomes we showed by treating cultures with chloroquine, which is known to affect lysosomal pH. Administration of 100μM or 300μM chloroquine for two hours resulted in a significant, much higher than after doxorubicin, dose-dependent decrease in SA-β-gal (Figure 5B). In parallel, we observed that chloroquine diminished neuronal staining with LysoTracker, which is a dye selective for acidic vacuoles (Figure 5C).

### SA-β-gal activity in aging mouse brains

**Figure 6:**
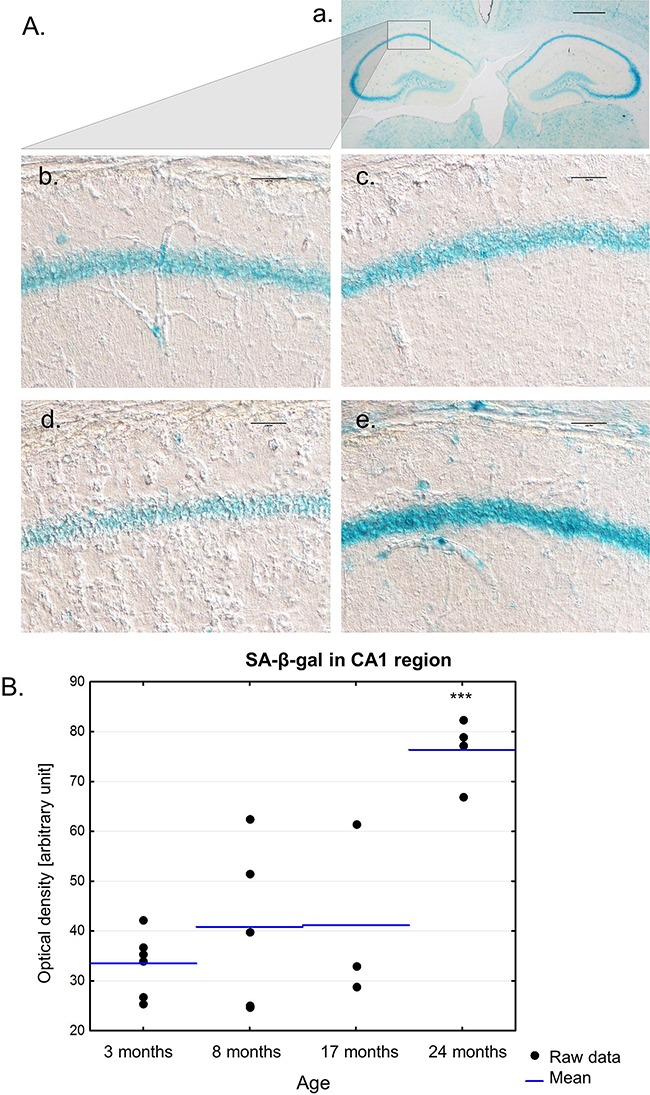
The presence of SA-β-gal activity in the hippocampus of mice of different ages **A.** A low magnification image of hippocampi with a neighbouring area in the brain of the 3-month-old mouse. Scale bar represents 500 μm (a). Representative images of SA-β-gal staining in the hippocampus of 3-month- (b), 8-month- (c), 17-month- (d), 24-month-old mice (e). Scale bars represent 100μm. **B.** Quantification of SA-β-gal staining in the hippocampus of mice of different ages. Single replicates are shown with blue lines representing the mean value, ***P>0.001 relative to all remaining groups (ANOVA).

As previously shown by Geng [9], SA-β-gal activity increases in the CA3 region of the hippocampus of aging rats. We stained brain sections of 3-, 8-, 17- and 24-month-old mice for SA-β-gal and, interestingly, we observed staining of the whole hippocampus in all groups. A representative image of hippocampi with the neighbouring brain area is presented in Figure 6A (a). The mean intensities of SA-β-gal in the CA1 region of the hippocampus in 3-, 8- and 17-old mice were comparable (Figure 6A (b, c, d, e), Figure 6B). However, whereas all young 3-month-old animals expressed SA-β-gal at a similar level, middle-aged mice experienced more variability in the intensity of SA-β-gal staining in the hippocampus. Significant rise in SA-β-gal was observed only for the oldest group of animals, which were 24 months of age. However, we are aware that the small number of animals in each age group did not allow for proper quantitative results.

## DISCUSSION

SA-β-gal is a commonly used biomarker of cell senescence. Cellular senescence is by definition an irreversible growth arrest of proliferating cells. However, recently it has been shown that in long-term culture of postmitotic neurons there was an increase in the number of SA-β-gal-positive cells, implying that some markers of replicative senescence can be also present in postmitotic cells. Accordingly, increased activity of SA-β-gal in rat cerebellar and hippocampal neurons and mouse neurons *in vitro* was revealed [9, 10, 17]. Pronounced elevation of the number of SA-β-gal- positive hippocampal neurons, reaching about 80% at 12 DIV, was observed, whereas in our cultures there were approximately 10% of SA-β-gal- positive neurons at the same DIV. In spite of this difference, it was characteristic of all of these studies, including ours, that SA-β-gal activity appeared quite early in cultured neurons, usually at 6-8DIV.

SA-β-gal assay measures the activity of a lysosomal β-galactosidase. Therefore, an increase in SA-β-gal could be due to increased enzyme activity or expression. Research suggests that the latter is the case. SA-β-gal activity in senescent dividing cells is owed at least in part to increased levels of lysosomal β-galactosidase mRNA and protein and has been linked to increased lysosome number or activity [18]. Moreover, it is likely that SA-β-gal is not directly linked to senescence because silencing of the *Glb1* gene, which encodes lysosomal β-galactosidase, does not alleviate the symptoms of senescence [18]. Thus, we think that rather than senescence we witnessed a continued growth of neurons, in which, as our experiments showed, lysosomes grew in number giving rise to increased SA-β-gal activity. However, it cannot be excluded that during development *in vitro* neurons can undergo a senescence-like process, which is characterized by similar markers as senescence of dividing cells. Accordingly, we analysed DNA damage in neurons. It is believed that DDR signalling has a causative role in the establishment of cellular senescence [19]. DNA damage is known to be involved in the induction of replicative senescence and premature senescence can be caused by such agents as ionizing radiation or DNA damage-inducing drugs [20, 21]. For example, in our previous studies we showed that low doses of doxorubicin added to cancer or normal cells induced SIPS, one of the features of which was increased SA-β-gal [22, 23]. In cortical neurons, however, we demonstrated minimal DNA double-strand damage during a long-term culture in both SA-β-gal positive and negative cells, which implies that neurons acquired SA-β-gal activity without DDR activation. Further, triggering DDR by low doses of doxorubicin did not increase the number of SA-β-gal-positive neurons. Altogether, we can conclude that markers, which in theory should label senescent-like neurons, namely SA-β-gal and DNA damage developed at a different time scale, which argues against any correlation between them.

We also studied other markers, which are linked to senescence of dividing cells: macroH2A present in senescence-associated heterochromatin foci (SAHF) and a cell cycle inhibitor p21. However, their pattern of expression appeared not to show any DIV-dependent changes (data not shown). We believe that examination of senescent-related cell cycle inhibitors to recognize senescence may not be relevant to postmitotic neurons, in which cell cycle regulators may perform different functions than in proliferating cells [24].

In order to shed more light on neuronal senescence *in vitro*, we studied the level of neuronal REST, which is a protein characteristic of aging brain and is known to promote expression of anti-apoptotic and antioxidant genes [12]. As far as we are concerned, this is the first observation of late induction of nuclear REST in neurons and we believe that we were able to identify a specific marker of neuronal aging *in vitro*. Neuronal REST upregulation occurred in cultures which also exhibited higher level of *IL-6* mRNA than early-DIV cultures. Increase in IL-6 indicates development of a pro-inflammatory environment which can foster pro-aging changes in cells. In the brain astrocytes are a primary source of IL-6 [25]. Therefore, in our long-term cultures *IL-6* mRNA upregulation could result from increasing number of astrocytes. However, neurons can produce IL-6 under some conditions [26] and it cannot be excluded that aging neurons could also contribute to the observed increase in *IL-6* mRNA.

As SA-β-gal-positive cells appeared in culture much earlier than REST-positive cells we think that, contrary to REST expression, SA-β-gal activity cannot be considered as a specific marker of their senescence-like state. However, there are some reports on SA-β-gal activity attributed to the senescence-like phenotype of neurons in aging mouse brain [5]. It was also demonstrated that the number of SA-β-gal-positive hippocampal pyramidal cells increased to about 50% in the CA3 region of the hippocampus of 24-month-old rats, whereas in 6-month-old animals there were only 14% of such cells [9]. Our study showed increase in the SA-β-gal staining in the hippocampus of 24-month-old animals and we think that the observed induction of SA-β-gal at this advanced age reflects expansion of lysosomes in presumably senescent neurons under conditions of oxidative and proteotoxic stress. In line with this, another study showed overexpression of genes associated with lysosomes during aging and it was hypothesised that this overexpression was a cellular adaptive response to accumulation of abnormal proteins with age [27]. Overall, induction of SA-β-gal activity could denote stress accumulation in senescent neurons of the aging brain. Surprisingly, our study of SA-β-gal staining during the entire lifespan of mice showed also noticeable presence of SA-β-gal in the hippocampus already in 3-month-old animals. This suggests that, similarly to *in vitro* studies, this marker may be linked to non-senescent situations even *in vivo*. We would like to stress that SA-β-gal staining can be positive even in brains of young, non-senescent mice, so using this marker for assessment of senescence *in vivo* should be done cautiously.

To summarize, we were able to show changes in the expression of SA-β-gal in long-term neuronal cultures and in the hippocampus of mice of different ages, which leads us to conclusion that SA-β-gal activity is not a specific marker of neuronal senescence. We also showed that SA-β-gal increase correlated with the expansion of lysosomes. Lysosomes could be essential not only for protein quality control and protection from damage but also for other cellular functions such as neuronal development. In contrast to SA-β-gal, REST exhibited a pattern of expression that correlated with neuronal age (DIV) and we consider it as a good marker of *in vitro* senescence of neurons.

## MATERIALS AND METHODS

### Reagents and antibodies

The following antibodies were obtained from commercial sources: mouse anti-MAP2 (1: 200, Sigma, M4403), rabbit anti-p53BP1 (1:500, Novus Biologicals, NB100-304), mouse anti-γH2AX (P-Ser139) (1: 500, Novus Biologicals, NB100-78356), mouse monoclonal anti-ATM (phospho S1981) (1: 500, Abcam, ab36810), rabbit anti CHK2 (phospho Thr68) (1:500, Novus Biologicals, NB100-92502), goat anti-REST/NRSF (1:100, Santa Cruz Biotechnology, sc-15118). Doxorubicin was purchased from Sigma.

### Primary cortical neuron isolation

Investigation has been conducted in accordance with the ethical standards laid down in the Declaration of Helsinki and with national and international guidelines, and has been approved by the I^st^ Ethical Committee in Warsaw, Poland. Animals used to obtain neurons for cell cultures were sacrificed by isoflurane overdose. Primary cortical cultures were prepared from embryonic day 18 (E18) rat brains. Embryos of either sex were used.

Embryos were decapitated and cerebral cortices were removed and dissociated by incubating in Earle's Balanced Salt Solution (EBSS, Worthington) containing papain (20 units /ml) and DNase (100 units /ml) and EDTA (0,5 mM) for 10min at 37°C. Following digestion the material was triturated avoiding bubbling and cell suspension was filtered through a 70 μm cell strainer. Next, dissociated cells were pelleted and resuspended in medium containing ovomucoid (10 mg/ml), a papain inhibitor. Intact cells were separated from cell debris by centrifugation through a single step discontinuous density gradient. Cell fragments accumulated at the interface between EBSS and the dense ovomucoid solution while dissociated cells pelleted at the bottom of the tube. Supernatant was discarded and the pellet was finally resuspended in Primary Neuron Basal Medium (Lonza) supplemented with NSF-1 (Neural Survival Factor-1, Lonza), L-Glutamine (Lonza) and Gentamicin with Amphotericin (Lonza) Cells were counted and plated as required on poly-D-Lysine coated plates. Half the volume of culture media was exchanged every 3 or 4 days. Cultures were stained with a neuronal marker: MAP2 (microtubule-associated protein 2).

### MTT

Neuronal cultures were treated with DNA damaging compound (doxorubicin) as required in 96-well plates. MTT (Thiazolyl Blue Tetrazolium Bromide; Sigma) was added to the wells at a final concentration of 1mg/ml for 3 hours at 37°C. Afterwards the medium was removed and 100μl of acidified (0.06N HCl) isopropanol was added. Absorbance was measured at 570 nm and 690 nm (reference wavelength) using a 96-well plate reader.

### LDH release

Neuronal cultures were treated with doxorubicin as required in 96-well plates. Lactate dehydrogenase (LDH) release was measured using a colorimetric kit from Promega (CytoTox 96R Non-Radioactive Cytotoxicity Assay, G1780) following manufacturer's instructions. 50 μl of the supernatant was transferred from each well to the corresponding wells of a new 96-well plate and 50 ul of a substrate was added. After 20 min incubation at room temperature STOP buffer was added and absorbance was measured at 490 nm.

### Hoechst staining

Neurons were fixed in 4% paraformaldehyde followed by Hoechst 33342 staining (1μg/ml in PBS) for 10min. After washing with PBS neurons were observed under a Nikon Eclipse Ti microscope.

### SA-β-gal staining

SA-β-gal staining was performed according to a protocol published by Dimiri *et al* [28]. Cells were fixed in PBS buffer containing formaldehyde and glutaraldehyde for 5 min followed by 3 washes. Next, cells were incubated in phosphate buffer pH 6.0 containing potassium ferrocyanure, potassium ferricyanure, NaCl, MgCl_2_, X-gal for 24 hours in 37°C. After incubation cells were washed 3 times in PBS followed by MAP2 and p53BP1 immunofluorescence.

### Immunofluorescence

Cells were fixed in 4% paraformaldehyde. Next, cells were permeabilized with 0.01% Triton X-100 and blocked in a blocking buffer (0.01% Triton X-100, 1.5% goat or donkey serum, 2% BSA in PBS) for 10 min, followed by incubation with primary antibodies, prepared in a blocking buffer, for 1-2 hours in a room temperature. After three washes in PBS, cells were incubated with secondary antibodies, followed by Hoechst staining and washes in PBS.

### Image acquisition

Specimens were visualized with a confocal laser scanning microscope Leica TCS SP8 using 63×/1.4 oil immersion lens. Fluorescence was excited using the 405-nm line from a pulsed laser (for Hoechst/DAPI) and a white light laser for other fluorochromes. Emitted light was captured using a hybrid Leica detector. Confocal z section stacks were collected at 0.39-μm spacing through the depth of the specimen. The final images represent a maximum projection along the z axis. Images in Figures 6 and 2 were acquired by a fluorescent microscope Nikon Eclipse Ti with 40x/0.6 or 20x/0.45 Nikon lenses. Fluorescence was excited by a metal halide lamp (Lumen 200) and, in the case of bright field images, a Hoffman contrast was applied. To merge the fluorescent (MAP2 and 53BP1) and transmitted-light image (SA-β-gal) the latter was reversed in colours and used as an additional red channel with the fluorescent image.

### Measuring fluorescence intensity of REST staining

The mean pixel intensity of fluorescence was determined using ImageJ software. The intensity was measured specifically in the nuclei area of MAP2-positive cells after subtracting the background.

### Lysotracker red staining

Neurons were cultured until a chosen day or treated witch chloroquine. Next, 100 nM LysoTracker Red DND-99 (Molecular Probes) and the Hoechst 33342 dyes were added to neuronal cultures and incubated for 45 min. Afterwards fresh medium was added and the images of stained cells were taken using fluorescent (Nikon) or Sp8 confocal (Leica) microscopes.

### Analysis of gene expression using real time RT-PCR

Total RNA was prepared using a RNeasy micro kit (Qiagen) and treated with DNase I, (Invitrogen) prior to cDNA synthesis. RNA concentration and quality was determined spectrophotometrically. RNA (0.5 μg) was reverse transcribed with GoScript™ Reverse Transcriptase (Sigma) using 0,5 μg random hexamer primers (Sigma), according to the manufacturer's instructions. Real-time quantitative PCR was carried out on 7900HT Fast Real-Time PCR System (Applied Biosystems) with qPCR SYBR Green reagents (Thermo Scientific) and a primer concentration of 0.5 μM. PCR conditions were standardized to 40 cycles with the primers for specific rat mRNA sequences:

IL-6 F: 5’ TAGTCCTTCCTACCCCAACTTCC 3’

IL-6 R: 5’ TTGGTCCTTAGCCACTCCTTC 3’

GAPDH F: 5’ CCATCTTCCAGGAGCGAGATC 3’

GAPDH R: 5’ GCCTTCTCCATGGTGGTGAA 3’.

### Preparation of brain sections and SA-β-gal staining

C57BL/6 female mice were intraperitoneally injected with Morbital (pentobarbital sodium 133,3 mg/ml + pentobarbital 26,7 mg/ml) and perfused. After transcardial perfusion with 4% paraformaldehyde whole brains were stored in 30% sucrose at 4°C. Coronal sections were prepared (40μm) using cryostat (Leica) and stained for SA-β-gal for 24 hours. Sections from the CA1 regions of the hippocampus were imaged (Nikon) and analysed using commercially available ImageJ software. The average intensity of staining was recorded from five fields of each CA1 region and the background was subtracted. Intensity of hippocampal SA-β-gal staining (arbitrary units) was compared between age groups.

### Statistics

Analysis was conducted with statistical analysis software (STATISTICA10). Comparisons between groups were performed with unpaired Student's *t*-test or ANOVA with Bonferroni correction. Two-tailed p< 0.05 was considered statistically significant.

## SUPPLEMENTARY MATERIALS FIGURES


