# Supplementary material for: Is senescence-associated β-galactosidase a marker of neuronal senescence?
Source: Oncotarget. 2016 Oct 19;7(49):81099–109. doi: 10.18632/oncotarget.12752 (PMC5348379; doi:10.18632/oncotarget.12752)
Supplement: Supplementary file 1 [file oncotarget-07-81099-s001.pdf]

Is senescence-associated β-galactosidase a marker of neuronal senescence?

SUPPLEMENTARY FIGURES

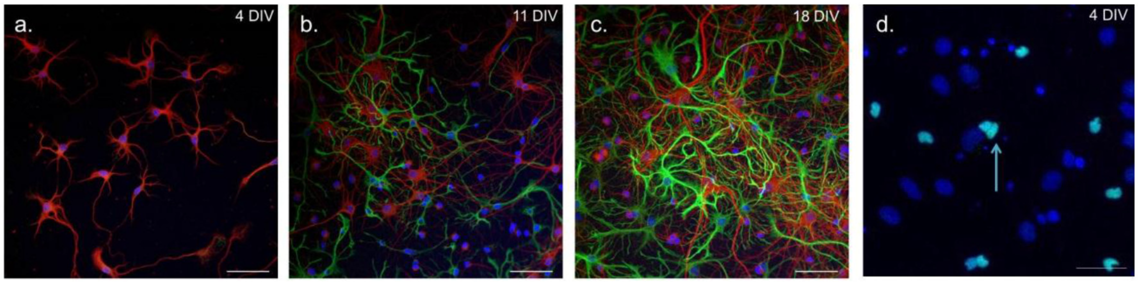

Supplementary Figure S1: A mixed neuronal-glial culture. The presence of neurons and glia in culture, as observed by expression of neuronal (MAP2, red) and astrocyte (GFAP, green) markers over time. 4 DIV **a.** 11 DIV **b.** 18 DIV **c.** Scale bars represent 50µm; The presence of oligodendrocyte precursors stained with Olig-2 antibody at 4 DIV (**d.** arrow). A scale bar represents 20µm.

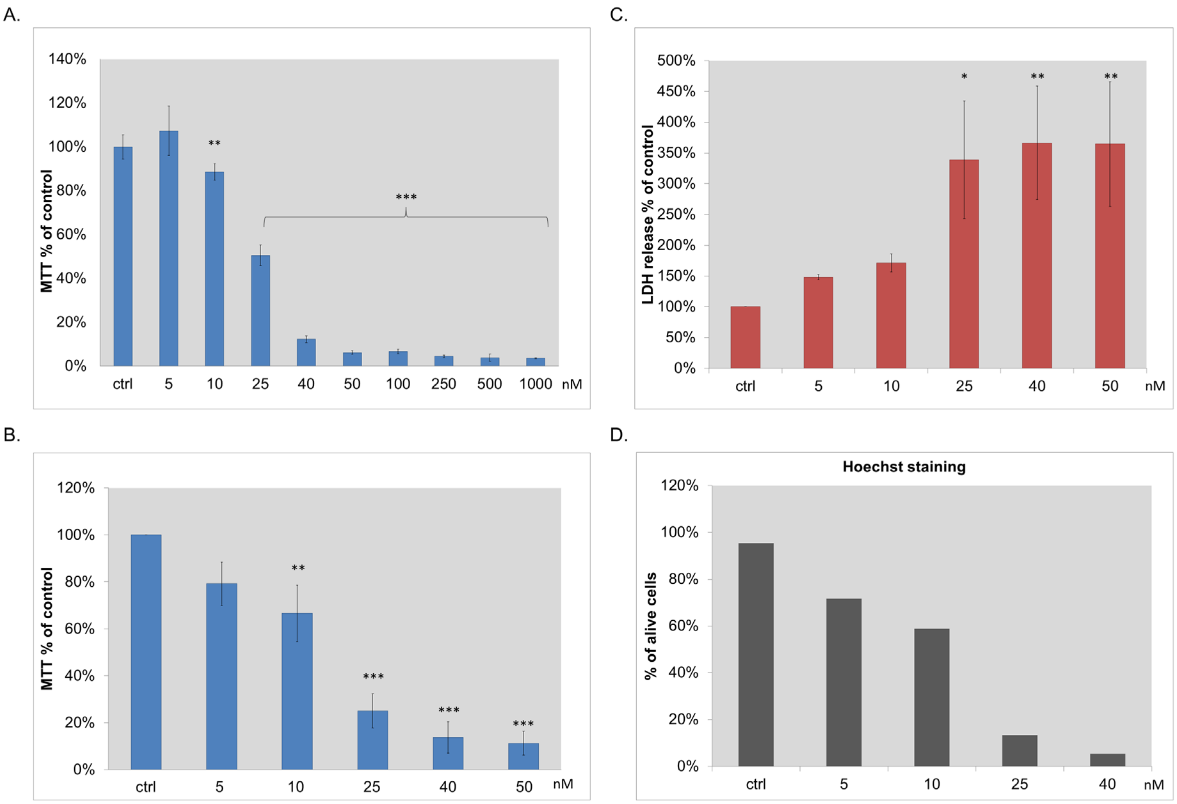

Supplementary Figure S2: Changes in cell viability upon doxorubicin treatment. A. 4 DIV cultures were treated with increasing doses of doxorubicin for 48 hours and MTT assay was performed. **B-D.** Neuronal cultures were treated with selected doses of doxorubicin for four days and viability was measured by MTT test (B), LDH release (C) or cells were fixed, stained by Hoechst 33342 and the percentage of apoptotic nuclei was counted (D). Values represent the mean ± S.D. from two independent experiments *P>0.05, **P>0.01, ***P>0.001 relative to control (ANOVA).
